# The Role of Bleomycin Sclerotherapy in Venous Malformation Management: A Narrative Review

**DOI:** 10.3390/life15101553

**Published:** 2025-10-03

**Authors:** Aikaterini Bini, Christos Topalidis, Triantafyllia Koletsa, Athanasios Papas, Efterpi Demiri, Leonidas Pavlidis

**Affiliations:** 1 Department of Plastic Surgery, School of Medicine, Aristotle University of Thessaloniki, Papageorgiou General Hospital, 564 03 Thessaloniki, Greece; drpapas@gmail.com (A.P.); depydemiri@gmail.com (E.D.); pavlidisl@yahoo.gr (L.P.); 2 Department of Pathology, School of Medicine, Aristotle University of Thessaloniki, 541 24 Thessaloniki, Greece; xtopalidis94@gmail.com (C.T.); tkoletsa@auth.gr (T.K.)

**Keywords:** bleomycin, sclerotherapy, venous malformations, vascular malformations

## Abstract

Venous malformations (VMs) are rare, non-involuting, slow-flow, congenital anomalies of vascular morphogenesis, presenting as dilated venous channels with reduced perivascular cell coverage. The treatment may be conservative or surgical, including laser therapy. The management of small superficial VMs typically involves surgical excision. In larger or deeper VMs, the intralesional–endovascular injection of the sclerosing agent bleomycin is the gold standard, as it eliminates the dysplastic venous vessels by inducing fibrosis and therefore promotes regression of the lesion. This review explores the current literature regarding the role of bleomycin in venous malformation management, emphasizing the molecular pathways involved, the efficacy of sclerotherapy with bleomycin and its complications and the associated management challenges. It evaluates the clinical and histological features of venous malformations, alongside diagnostic methodologies and treatment strategies, drawing on the most recent bibliographic data. The literature was systematically reviewed using the PubMed database, offering insights into future research directions and highlighting innovative treatment approaches.

## 1. Introduction

Venous malformations (VMs) are among the most common types of vascular anomalies, affecting roughly 1% of the population and accounting for 50–66% of all vascular malformations with an incidence of 1 to 2 cases per 10,000 [1,2]. They are congenital, slow-flow lesions composed of dysplastic venous channels that enlarge over time and cause symptoms such as swelling, pain, bleeding and functional or cosmetic impairment [1,2]. VMs can present anywhere in the body, from the extremities and trunk to the head and neck, including the tongue and orbit. They are often visible at birth or soon after birth, but they may manifest clinically during childhood or early adulthood [2,3]. In anatomically and functionally sensitive areas like the tongue, orbit and airway, these lesions can significantly impact quality of life and may require urgent management [4,5,6,7,8].

The International Society for the Study of Vascular Anomalies (ISSVA) classifies VMs as slow-flow, simple malformations and the management typically begins with conservative measures [9]. Compression garments, particularly for limb lesions, are the first-line treatment to reduce symptoms when the lesion is accessible and compressible [3]. When conservative management fails or the lesions become symptomatic, percutaneous sclerotherapy is the treatment of choice in the majority of cases. Other interventions like laser ablation, surgical excision or embolization may also be suitable depending on the size, location and flow characteristics [3].

Bleomycin, originally an antitumor antibiotic, has emerged as a prominent sclerosant. The idea of using bleomycin as a sclerosing agent for vascular malformations originated in 1977, when intralesional bleomycin was injected for lymphatic malformations/cystic hygromas and later on for venous malformations. Intralesional bleomycin induces fibrosis by damaging endothelial cells and recruiting fibroblasts, leading to lesion regression with minimal pronounced post-sclerotherapy swelling compared to other agents like ethanol. It is often used under ultrasound or fluoroscopic guidance, with doses typically ranging from 0.5 to 1 mg/kg up to around 15 mg per session [10].

The purpose of this study is to review the current literature regarding the role of bleomycin sclerotherapy in venous malformation management, including complications, molecular pathways and promising innovative approaches.

## 2. Materials and Methods

The literature was reviewed using the PubMed database, with ‘bleomycin’ and ‘venous malformations’ as the main search terms. Citations of papers found by the initial search were also used to find references. Articles including other vascular anomalies apart from venous malformations and articles including other sclerosing agents apart from bleomycin were excluded. Articles referring to the combination of bleomycin with other sclerosing agents (e.g., polidocanol and sodium tetradecyl sulfate) or articles comparing the efficacy and complications of bleomycin to other sclerosing agents were included. In total, 49 of the 107 original English-language articles, reports and reviews between 2015 and 2025 were finally included, offering insights into current and future research directions and highlighting innovative treatment approaches (e.g., bleomycin electrosclerotherapy), based particularly on the mechanism of action of bleomycin at histological and molecular level. Regarding the molecular pathways involved in the action of bleomycin, the literature search was carried out in a similar manner. Specifically, the PubMed database was searched using the terms ‘bleomycin’, ‘venous malformations’ and ‘molecular pathways’, which yielded a review on orbital venous-lymphatic malformations, without any mention of bleomycin’s action on a specific molecular pathway. Therefore, citations in the papers found by the initial search were used to find references.

## 3. Results

### 3.1. Sclerotherapy with Bleomycin Based on the Anatomical Site of the Lesion

Percutaneous sclerotherapy with bleomycin is a widely accepted treatment for head and neck venous malformations, achieving subjective or objective reduction of lesion size in approximately 96% of cases, with a low major complication rate of around 1.1%, including significant swelling due to venous thrombosis, which may obstruct the eyesight or the airway depending on the anatomical area of the primary lesion [5,11]. Intralesional bleomycin has been effectively used for peri-ocular venous malformations, with studies showing favorable outcomes in eyelid and orbital venous lesions, often requiring multiple sessions (1 to 6), causing minimal local side effects such as transient swelling or erythema [5,11].

A retrospective study of tongue VMs (36 patients, 140 procedures) reported symptomatic improvement in 93.8% of treatments and imaging-confirmed reduction in 64.3% of evaluable cases; side effects were minimal, mostly involving transient swelling [4]. Systematic reviews show subjective reduction in 96% and objective shrinkage in over 90% of vascular malformations of the head and neck, with low rates of minor adverse events and no reported major complications like pulmonary fibrosis [12].

In solid venous malformations of the lower extremities, resistant to foam sclerosants, interstitial bleomycin can reduce pain in 92% of cases and resolve intralesional vascular flow on Doppler ultrasound, with only minor side effects like skin pigmentation and allergic reactions [11,13]. 

A broader review of percutaneous bleomycin sclerotherapy in mixed vascular anomalies reported significant improvement in 82% of venous malformations, with tolerable acute side effects; mainly pain and headache in about 23% of cases [11].

### 3.2. Bleomycin Forms and Pharmacokinetics

Bleomycin has long been used as a liquid sclerosant, most commonly in percutaneous/intralesional injections, exploiting its DNA-cleaving mechanism to induce endothelial damage and fibrosis in vascular malformations [14].

When administered intramuscularly or subcutaneously, bleomycin achieves peak plasma levels within 30–60 min, with systemic bioavailability reaching approximately 100% via intramuscular and 70% via subcutaneous routes, while renal clearance is the primary elimination route; around 65% of the intravenous dose is excreted in urine within 24 h [14]. As a sclerosing agent, bleomycin is delivered intraluminally or interstitially in vascular malformations, demonstrating a terminal elimination half-life of roughly 88–112 min, modest distribution volumes of 1.5–4.9 L and measurable systemic exposure with an area under the curve (AUC) of 54–129 mg min/L, confirming systemic absorption even with local delivery [14].

In clinical use, dose limits of around 15 mg per session are adequate and can mitigate the risk of pulmonary fibrosis, especially since bleomycin hydrolase is sparse in lung and skin tissues. More recently, bleomycin has been formulated into foam, either alone or in combination with polidocanol, in order to enhance sclerosing efficacy by increasing the surface area and the contact time with the vessel walls [15]. Bleomycin–polidocanol foam (BPF) is prepared by the Tessari method (liquid–air 1:4, mixed via syringes), exhibiting a substantially prolonged half-life (up to ~246 s), reduced bubble diameter and improved stability in vitro compared to polidocanol or bleomycin alone [15].

Optimization studies show that dissolving bleomycin in human serum albumin (HSA) without saline and using a 3:1 air–liquid ratio yields foam with a half-life around 7.5–9 min [15]. While any further addition of bleomycin enhances foam stability, varying concentrations beyond a threshold may not further prolong stability [15]. Clinically, BPF yields favorable outcomes, achieving average lesion volume reduction of ~78% and significant pain relief in venous malformation patients, with acceptable safety profiles [15].

In summary, both liquid and foam forms of bleomycin are viable sclerosants: the liquid form offers predictable systemic absorption and pharmacokinetics, while foam modalities (especially when combined with polidocanol and albumin) enhance local efficacy via improved stability and contact. Nevertheless, pharmacokinetic profiles remain similar in terms of absorption and elimination when used intralesionally.

### 3.3. Complications

Sclerotherapy with bleomycin, although generally safe, carries a risk of both local and systemic complications when used to treat venous malformations. Mild to moderate pain and transient swelling are the most frequently reported adverse effects, occurring in approximately 23–30% of patients, typically resolving within 24 h without intervention [11,12,16]. Local skin hyperpigmentation and transient erythema have been documented in up to 10% of cases, reflecting bleomycin’s inflammatory impact on superficial tissues. Skin blistering, ulceration or necrosis are infrequent but possible, especially when treating superficial or poorly compressible lesions, necessitating careful technique to avoid extravasation. Rarely, patients may experience minor allergic reactions, manifesting as local urticaria or mild systemic rash, with only a small minority requiring antihistamines. [11,17]. Patients with mild allergic reactions to bleomycin may be suitable for pre-medication (antihistamines, corticosteroids and antipyretics) and re-administration of bleomycin at a slower rate and/or at a lower dose under close monitoring. The decision for bleomycin re-administration after mild allergic reaction should be individualized and based on the patient’s general condition and co-morbidities as well as the capacity of each center to provide intensive care in case of a life-threatening reaction. In case of severe anaphylactic reaction, bleomycin treatment should be permanently discontinued.

More serious local effects include transient neurologic symptoms such as sensory or motor deficits; these have been reported following sclerotherapy of lesions near nerves, though they typically resolve over weeks to months. Coagulopathy and consumption of clotting factors have been very rarely observed, particularly when concomitant extensive sclerotherapy is performed or when bleomycin is used in combination with other sclerosants like ethanol [3].

Interstitial bleomycin injections targeting lower extremity solid venous malformations have occasionally caused minor allergic reactions and residual pigment changes, though no major adverse events have been noted [3,11,13].

Systemic complications like pulmonary fibrosis have rarely been observed in localized treatment, while bleomycin levels remain undetectable in the bloodstream after intralesional injection [18]. Bleomycin-related pulmonary toxicity (e.g., fibrosis) remains extraordinarily rare at intralesional doses, with only sporadic case reports of acute pulmonary inflammation in infants receiving ≥7 mg, while no long-term lung injury has been reported in larger series [18]. Routine laboratory monitoring typically shows no clinically significant hepatic, hematologic or renal abnormalities following bleomycin sclerotherapy [19].

Overall, sclerotherapy with bleomycin is a safe and effective option across various anatomical regions, including the head and neck, extremities and orbital area, offering high lesion control and low rates of serious adverse effects. While bleomycin sclerotherapy for venous malformations is associated mostly with mild, self-limited local effects, vigilance is warranted to prevent rare but serious complications, particularly when treating lesions near critical structures or in pediatric patients [11,12,13,16,17,18].

### 3.4. Outcomes and Affecting Factors

Overall, satisfying results have been reported in the literature. Previous single-center series found sclerotherapy beneficial in 82–90% of VM cases and tolerable overall, with manageable side effects such as pain and headache in under a quarter of patients [10,11]. A prospective study of 50 patients (April 2020–March 2022) treated with ultrasound-guided and fluoroscopy-guided intralesional bleomycin found objective improvement in 79.5% of cases and reported no major complications; diffuse lesions were the only independent predictor of poor response [10].

Bleomycin sclerotherapy has become a cornerstone in the management of venous malformations (VMs), with clinical studies consistently demonstrating significant efficacy and high patient satisfaction. In a large retrospective cohort of 138 VM patients treated with bleomycin–polidocanol foam (BPF), mean lesion volume reduction approximated 78.5% alongside marked pain relief; 70.3% of patients reported symptom resolution after a single session, while minor complications were recorded in 22.5% of cases [20].

Percutaneous bleomycin sclerotherapy combined with sodium tetradecyl sulfate (STS) administered to 35 patients across head, neck and extremity VMs showed complete obliteration in 16 cases and a significant (>50%) shrinkage in another 13, with no recurrence over a follow-up of up to three years [21]. Furthermore, a mid-term follow-up of 15 patients with VMs revealed ≥50% volume reduction in 64% of VMs, alongside improvement in pain, cosmesis and overall patient satisfaction, with minor complications and no serious adverse events [16]. Another retrospective review involving 17 patients with VMs reported significant improvement in 82.4% of VM lesions after an average of 3.2 sessions, with transient side effects like pain and headache in 23.3% of the cases [11].

In the current literature, there is no significantly superior sclerosing agent in terms of effectiveness. Systematic reviews and meta-analyses reinforce bleomycin’s curated placement among sclerosants; whereas ethanol offers slightly higher efficacy (up to 95%) with fewer sessions but with higher complication rates, bleomycin demonstrates more modest volume reductions (43–82.7%) with lower adverse event frequency [3,16,18,22,23]. Its reduced toxicity profile, limited mainly to transient hyperpigmentation and fever, renders it ideal for delicate anatomical regions, such as the head and neck [24]. Overall patient-reported satisfaction rates over around 82%, with clinical response to swelling being the best predictor of contentment [11].

Several key factors influence bleomycin’s therapeutic outcomes. Lesion angioarchitecture plays a pivotal role: simple VMs with minimal drainage (Puig types I-II) retain sclerosant longer and show better responses, whereas lesions with rapid outflow (Puig types III-IV) tend to resist treatment [11]. Foam formulation and combination strategies enhance efficacy: combined polidocanol and bleomycin foam achieved a 93.3% clinical success rate at six months in over 210 patients, suggesting synergistic interaction between agents [25]. Patient parameters such as high initial pain score and elevated APTT correlate with superior pain relief after sclerotherapy, while severe initial pain and high thrombocytocrit may predict incomplete post-sclerotherapy pain resolution [20]. Ultrasound or fluoroscopic guidance during injection allows precise agent delivery, reducing extravasation and enhancing lesion targeting, thus improving volumetric outcomes and minimizing complications.

Adverse event risks are relatively low: Minor complications such as swelling, pain, hematoma and skin pigmentation affect 20–30% of patients, while serious complications and pulmonary fibrosis remain exceptionally rare with intralesional doses within safety margins (≤15 mg per session). However, age below 18 and skin involvement increase the likelihood of cutaneous reactions [11].

Overall, lesion characteristics (size, drainage and morphology), sclerosant formulation, imaging-guided delivery and individual patient factors (coagulation parameters, baseline symptoms and anatomical region) shape bleomycin’s final outcomes, indicating a multifactorial interplay that underpins its consistent safety and efficacy in VM management.

### 3.5. Sclerotherapy with Bleomycin in Pediatric Population

Bleomycin has become a widely accepted sclerosant for the treatment of pediatric VMs due to its potent cytotoxic action against vascular endothelium and supporting stromal cells, with limited swelling compared to alternatives like ethanol or doxycycline [24,26]. In pediatric patients, foam formulations of bleomycin, often produced via the Tessari method, have demonstrated robust efficacy, reducing lesion volumes by approximately 66% on average, while requiring fewer injections and lower total doses than the liquid form [24]. A randomized controlled trial in children with cervicofacial VMs found that foam bleomycin achieved over 90% lesion volume reduction in two-thirds of cases, with fewer sessions and lower cumulative doses compared to liquid bleomycin, indicating enhanced efficiency and safety [24]. Furthermore, clinical experience in younger patients under general anesthesia highlights bleomycin’s low risk of neurotoxicity and minimal post-procedural edema, making it particularly suitable for delicate anatomical regions [24,26].

Retrospective reviews including over 200 patients (mean age: 25 years, including the pediatric cohort), using combined polidocanol foam and liquid bleomycin therapy, have reported high clinical efficacy (>93% overall), with bleomycin playing a central role in achieving effective sclerosis of slow-flow VMs [25]. Interestingly, meta-analyses covering more than 1300 pediatric and young-adult cases have shown VM size reduction rates of 84–87%, with significantly fewer adverse events and no pulmonary fibrosis incidents after intralesional injection of bleomycin [18]. Studies suggest bleomycin volume reduction of ≥50% in over 64% of VM cases, accompanied by rare minor complications such as transient swelling, hyperpigmentation [24,26] or superficial skin infection, skin necrosis, hyperpigmentation and minor contour deformity [27]. Life-time dose limits are strictly observed (usually <15,000 IU per session), with no documented pulmonary toxicity in large cohorts [17,18,28].

Overall, evidence supports intralesional bleomycin, especially foam formulations, as a safe, effective first-line sclerosant for pediatric VMs, combining high efficacy, minimal systemic exposure and low complication risk when administered within recommended dosing guidelines [27].

### 3.6. Bleomycin Electrosclerotherapy (BEST)

Bleomycin electrosclerotherapy (BEST), which combines intralesional bleomycin with reversible localized electroporation, shows promise in enhancing efficacy in lesions resistant to standard sclerotherapy, by facilitating endothelial uptake and lesion collapse, achieving up to 86% volume reduction with fewer sessions. BEST can significantly enhance the cytotoxic effect on vascular malformation endothelial walls by temporarily increasing cell membrane permeability [29,30,31,32]. Physiologically, electroporation induces a “vascular lock” that restricts blood flow and boosts intracellular bleomycin concentration by up to several hundred- to thousand-fold, thereby reducing systemic exposure while increasing local efficacy [29,30,32]. First described in 2017 for refractory venous malformations, BEST has since been shown to achieve marked clinical improvements even with lower bleomycin dosages than standard sclerotherapy [32].

In a prospective series of 17 patients with VMs, BEST produced complete or significant responses in approximately 80% of cases, with mild transient swelling, pain and bleeding as the most common side effects [31]. A retrospective case series, published in 2021, involving 17 patients with sclerotherapy-resistant VMs reported a dramatic median volume reduction of 86% (from ~25 cm^3^ to ~3.5 cm^3^) after a single session and low bleomycin doses (~3 mg), with sustained symptom relief [29]. A larger multicenter cohort that included adults and children reported similar findings, showing significant reduction of lesion size with fewer sessions, improved mobility and cosmesis along with reduced pain. Skin hyperpigmentation was the most frequent side effect, although it was generally mild [33].

Pediatric and adolescent patients demonstrate especially favorable outcomes compared to adults, with higher rates of symptom improvement and lower complication burdens [33]. An international working group under the name International Network for Sharing Practices on Electrochemotherapy (InspECT) noted the absence of standardized BEST protocols and emphasized the need for clinical trials to refine optimal drug dosing, electroporation parameters, electrode selection, anesthetic approaches and safety monitoring [30].

Overall, current evidence supports BEST as a promising, efficacious and generally well-tolerated treatment for VMs, particularly in refractory or pediatric cases. Nevertheless, further standardization and controlled trials are required to establish best practices.

### 3.7. Molecular Signaling Pathways in Venous Malformations and How Bleomycin Affects Them

Coordinated efforts over the past twenty years have elucidated the molecular landscape of vascular anomalies (VAs). As a result, more accurate classification schemes, more efficient management and targeted therapies are now available. The major signaling pathways that govern the development of these anomalies include (a) RAS (rat sarcoma)/RAF (rapidly accelerated fibrosarcoma)/MAPK (mitogen-activated protein kinase)/ERK (extracellular signal-regulated kinase); (b) angiopoietin/TIE2 (angiopoietin-1 receptor) and PI3K (phosphoinositide 3-kinase)/AKT (protein kinase B)/mTOR (mammalian target of Rapamycin); (c) TGF-β (transforming growth factor beta) signaling and (d) the G protein–coupled receptor signaling molecules (GNA [G protein subunit alpha] Q/GNA11/GNA14) [34,35].

The PI3K/AKT/mTOR pathway is primarily associated with the slow-flow vascular malformations, namely lymphatic and venous malformations [34]. Venous malformations (VMs), like any kind of vascular anomaly, can be subclassified from a genetic standpoint as sporadic and hereditary (Table 1).

Since the comprehensive review of the molecular background of the various venous malformations is beyond the scope of this review, this study focuses on common venous malformation (CVM).

Approximately 50% of CVMs are related to TEK and 20–25% to PIK3CA gain-of-function mutations [36,37]. The remaining cases are associated with mutations in other genes of the PI3K/AKT and MAPK/ERK signaling pathways [34,38,39]. Both TEK and PIK3CA genes are involved in the pathogenesis of VMs via the activation of various components (AKT, STAT1 and ERK1/2) of the PI3K/AKT/mTOR pathway [38]. The TEK gene (located on chromosome 9p21.2) encodes the endothelial receptor tyrosine kinase TIE2. When its ligand, namely angiopoietin 1 (ANPT1), binds, the canonical PI3K-AKT-mTOR pathway is activated, leading to endothelial cell proliferation. More than 20 different mutations are described in the literature, all of which are in the intracellular domain of the receptor, with the most common detected being TEK L914F. The PIK3CA gene (located on chromosome 3q26.32) encodes the alpha subunit of the downstream effector PI3K, which is activated by the VEGF and TIE receptors to eventually phosphorylate AKT [35,39].

The underlying gene mutations that contribute to the development of the VAs seem to participate both in the impaired development and function of endothelial cells (ECs), vascular smooth muscle cells (VSMCs) and proteins of the extracellular matrix (ECM) [35,38]. VMs are characterized by an attenuated layer of ECs, an intermittent cover by VSMCs and a disorganized ECM.

A major player that orchestrates the deposition and stabilization of ECM is lysyl oxidase (LOX). Its role also extends to vascular homeostasis, cardiovascular development, fibrotic and cardiovascular diseases and cancer progression. LOX expression is regulated by various growth factors and cytokines, with the major role belonging to TGF-β [40,41]. Zhu et al. demonstrated that LOX expression was significantly reduced in VM tissues, in contrast to normal skin. Furthermore, they confirmed the attenuated layer of VSMCs in VM tissues, which led to the assumption that the decreased LOX expression is responsible for the vascular destabilization [42].

Dysregulation of the ECM is also influenced by microRNA-21 (miR-21), a small noncoding RNA with profibrotic properties implicated in the abnormal deposition of collagen, which is essential for vascular morphogenesis and stabilization. Zhu et al. demonstrated a significantly decreased expression of miR-21, accompanied by reduced collagen deposition in VM samples compared to normal vessels. The authors were able to identify a causative relation between miR-21 and collagen expression. The former is regulated, at least partially, by the TGF-β/Smad3 pathway in a positive feedback loop [43].

MicroRNAs are also implicated in the impaired function of VSMC, with miR-145 constituting a typical example. However, an underlying signaling pathway regulating miR-145 function is yet to be uncovered, apart from its relationship with TGF-β [44].

VMs are associated with developmental deficits during the complex process of angiogenesis, leading to the formation of dilated and dysmorphic venous channels [35]. Venous stasis and thrombosis at the site of a VM are well-characterized features that create a hypoxic environment, which is known to induce angiogenesis. A key regulator in response to hypoxic/ischemic signals is the transcription factor hypoxia-induced factor-1α (HIF-1α). In its stable form, HIF-1α is translocated to the nucleus, where it dimerizes with HIF-1β to induce the expression of various genes related to hypoxia adaptation, angiogenesis and glucose metabolism. HIF-1α is subjected to regulation by the PI3K/AKT/mTOR and PI3K/AKT/FRAP signaling pathways, since it has been demonstrated that PI3K/mTOR inhibitors suppress the expression of HIF-1α [45]. The interplay between hypoxia and angiogenesis has led to the hypothesis that during embryogenesis, a hypoxic background could induce the expression of HIF-1α and therefore the upregulation of angiogenesis with the probable formation of a VM in the appropriate genetic substrate. Chung et al. demonstrated immunohistochemically the increase of HIF-1α expression in VM tissue compared to normal samples in a hypoxic environment. This result was also confirmed on the mRNA level, highlighting the fact that VMs are more susceptible to hypoxia signals than normal vessels [46].

Unveiling the genetic environment that governs VM’s formation could eventually lead to targeted treatments. Many clinical trials targeting the PI3K/AKT/mTOR pathway are showing promising results [34,35,38,39]. Treatment modalities currently include conservative therapy, embolization, ablation, sclerotherapy, laser treatment and surgery and are tailored according to the patient. Sclerotherapy, due to its minimal invasiveness, is usually the preferred choice. Various agents are available, and among them, bleomycin is commonly used due to its safety profile in terms of adverse events [1,3,18].

The mechanism for bleomycin’s action in the context of VM treatment seems to include endothelial damage, acute and chronic inflammation and fibrosis [18,47]. The latter is accompanied by endothelial-to-mesenchymal transition (EndoMT) in a manner analogous to the well-known epithelial-to-mesenchymal transition (EMT), which is involved in various processes and especially in cancer invasion and spread. Zhang et al. used human umbilical vein endothelial cells (HUVECs) cultured with bleomycin, revealing dramatic changes in cell morphology, a switch to a mesenchymal phenotype at both the immunohistochemical and mRNA levels, impaired tube formation and enhanced migration ability, strongly suggesting that bleomycin treatment induces EndoMT. According to their research, this process is under the regulation of Slug, a member of the zinc finger transcription factor family. Slug is modulated, among others, by the AKT/mTOR signaling pathway and bleomycin seems to activate AKT and its downstream signals, as demonstrated by the study. Intriguing is also the fact that their results were confirmed in VM samples from patients treated with bleomycin [48].

As stated above, LOX expression is significantly suppressed in VMs. To identify its potential clinical significance, Zhu et al. studied HUVECs cultured with bleomycin and demonstrated an increase in mRNA expression of LOX with upregulation of various ECM components [42]. Sclerotherapy also increased the expression of TGF-β, which is also said to be implicated in the process of EndoMT. TGF-β-mediated EndoMT involves several signaling pathways (e.g., Smad, MAPK/ERK, PI3K and p38 MAPK), which are also implicated in the pathogenesis of VMs [42,48]. The role of TGF-β is also highlighted in the work of Zhu et al., where it was demonstrated both in VM samples and HUVECs treated with bleomycin the upregulation of TGF-β/Smad3/miR-21 axis, which seems to play an important role in collagen expression and deposition [43]. Upregulation of TGF-β and miR-145 has also been revealed with administration of bleomycin [44].

An alternative pathway leading to fibrosis following bleomycin’s administration is mediated through a distinct form of programmed cell death called pyroptosis. This includes the increase in cell volume, the formation of pores in the cell membrane and the release of proinflammatory cytokines. The process is regulated by the NLRP3/caspase-1/GSDMD pathway, which is activated following bleomycin treatment, as highlighted by Chen et al. [47].

**Table 1:** Genetic subclassification of the most common venous malformations (VMs). In this list, vascular anomalies (VMs included) associated with other anomalies are not mentioned [9,34,35,49].

Sporadic	Molecular Background	Hereditary	Molecular Background
Common Venous Malformation (CVM) (94% of the total cases of VMs)	TEK-activating mutations PIK3CA-activating mutations	Mucocutaneous Venous Malformation (VMCM)	TEK-activating mutations
Multifocal Sporadic Venous Malformation (MSVM)	TEK-activating mutations	Glomuvenous malformation (GVM)	GLMN loss-of-function mutations
Blue Rubber Bleb Naevus syndrome (BRBN)	TEK-activating mutations	Familial Intraosseous Vascular Malformation (VMOS)	ELMO2 loss-of-function mutations
Cerebral Cavernous Malformation (CCM)	MAP3K3-, MAP2K7-, PIK3CA-activating mutations	Cerebral Cavernous Malformation (CCM) (20% of cases of CCM)	KRIT1, CCM2, PDCD10 loss-of-function mutations
Verrucous Venous Malformation (VVM)	MAP3K3-activating mutations		

## 4. Conclusions

In summary, intralesional bleomycin is a well-tolerated and effective treatment for slow-flow venous malformations, especially in delicate anatomical regions. It offers a high response rate (≈80–95%), having a favorable safety profile and minimal side effects. Bleomycin electrosclerotherapy may further enhance outcomes in resistant lesions. The most consistent predictors of reduced response include lesion diffuseness and extensive involvement. Adherence to dose limitations and proper image-guided techniques is the key to minimizing risk. Continuous studies, particularly randomized and long-term follow-up results, will further refine bleomycin’s role in VM management. In the future, targeted therapies may also obtain a more active and even the predominant role in venous malformation management.

## Data Availability

The data supporting the conclusions of this article may be available by the authors on request.
